# Direct Inhibition of IRF-Dependent Transcriptional Regulatory Mechanisms Associated With Disease

**DOI:** 10.3389/fimmu.2019.01176

**Published:** 2019-05-24

**Authors:** Aleksandra Antonczyk, Bart Krist, Malgorzata Sajek, Agata Michalska, Anna Piaszyk-Borychowska, Martyna Plens-Galaska, Joanna Wesoly, Hans A. R. Bluyssen

**Affiliations:** ^1^Department of Human Molecular Genetics, Faculty of Biology, Institute of Molecular Biology and Biotechnology, Adam Mickiewicz University, Poznań, Poland; ^2^Laboratory of High Throughput Technologies, Faculty of Biology, Institute of Molecular Biology and Biotechnology, Adam Mickiewicz University, Poznań, Poland

**Keywords:** IRF, interferon, TLR, transcriptional regulation, inflammation, inhibition

## Abstract

Interferon regulatory factors (IRFs) are a family of homologous proteins that regulate the transcription of interferons (IFNs) and IFN-induced gene expression. As such they are important modulating proteins in the Toll-like receptor (TLR) and IFN signaling pathways, which are vital elements of the innate immune system. IRFs have a multi-domain structure, with the N-terminal part acting as a DNA binding domain (DBD) that recognizes a DNA-binding motif similar to the IFN-stimulated response element (ISRE). The C-terminal part contains the IRF-association domain (IAD), with which they can self-associate, bind to IRF family members or interact with other transcription factors. This complex formation is crucial for DNA binding and the commencing of target-gene expression. IRFs bind DNA and exert their activating potential as homo or heterodimers with other IRFs. Moreover, they can form complexes (e.g., with Signal transducers and activators of transcription, STATs) and collaborate with other co-acting transcription factors such as Nuclear factor-κB (NF-κB) and PU.1. In time, more of these IRF co-activating mechanisms have been discovered, which may play a key role in the pathogenesis of many diseases, such as acute and chronic inflammation, autoimmune diseases, and cancer. Detailed knowledge of IRFs structure and activating mechanisms predisposes IRFs as potential targets for inhibition in therapeutic strategies connected to numerous immune system-originated diseases. Until now only indirect IRF modulation has been studied in terms of antiviral response regulation and cancer treatment, using mainly antisense oligonucleotides and siRNA knockdown strategies. However, none of these approaches so far entered clinical trials. Moreover, no direct IRF-inhibitory strategies have been reported. In this review, we summarize current knowledge of the different IRF-mediated transcriptional regulatory mechanisms and how they reflect the diverse functions of IRFs in homeostasis and in TLR and IFN signaling. Moreover, we present IRFs as promising inhibitory targets and propose a novel direct IRF-modulating strategy employing a pipeline approach that combines comparative *in silico* docking to the IRF-DBD with *in vitro* validation of IRF inhibition. We hypothesize that our methodology will enable the efficient identification of IRF-specific and pan-IRF inhibitors that can be used for the treatment of IRF-dependent disorders and malignancies.

## Introduction

**Figure 1:**
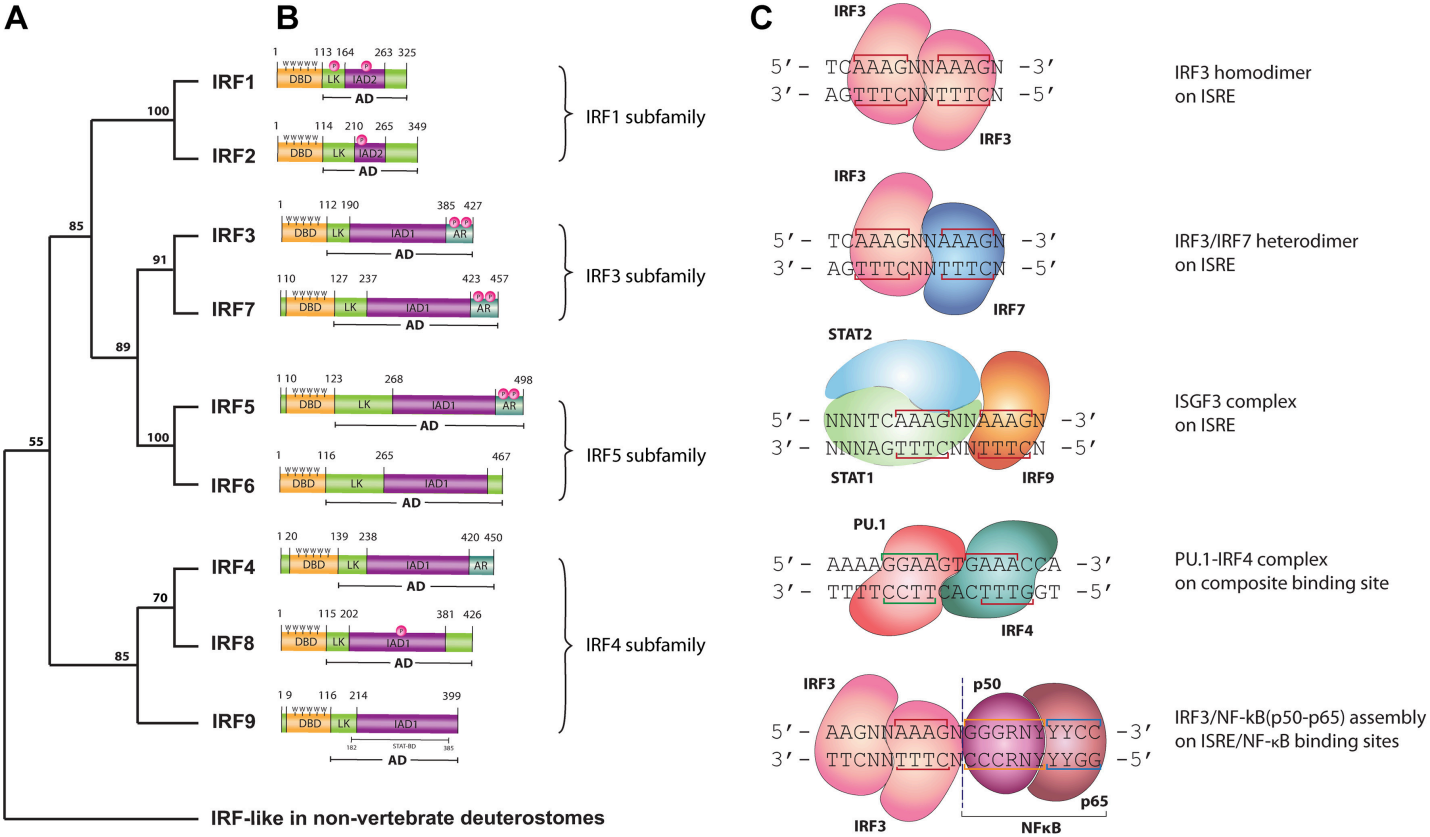
**(A)** Phylogenetic tree of the DNA-binding domain of IRF family proteins in vertebrates. Homologous protein sequences were searched using the NCBI BLAST server and aligned using ClustalW. Phylogenetic analyses were performed using the Neighbor-Joining method within the Mega 7.0 program. Data were analyzed using Poisson correction, and gaps were removed by pairwise deletion. The bootstrap values of the branches were obtained by testing the tree 10,000 times. Bootstrap values larger than 70% are shown next to the branches based on 10,000 replications. **(B)** Functional domains of human IRF proteins. DBD, DNA-binding domain; AD, activation domain; LK, linker region; IAD, IRF association domain type 1 (IAD1) or type 2 (IAD2); AR, auto-inhibitory region; P, phosphorylation site; 5W, five tryptophan repeats—“tryptophan cluster”; STAT-BD, STAT-binding domain. **(C)** DNA binding modes of IRFs. LINE—nucleotides involved in interaction with IRF—DBD; N, any nucleotide; R, purine; Y, pyrimidine. IRF3 homodimer, IRF3/IRF7 heterodimer and ISGF3 are bound to the consensus ISRE sequence with two ISRE half-sited “GAAA.” IRF4/PU.1 complex bind to the composite binding site, while NF-κB binds κB DNA element.

In 1988 the first interferon regulatory factor (IRF) was identified and named IRF1 (1, 2). Since then, a total of nine IRFs (IRF1-9) have been characterized in mammals. Recently, the presence of IRF10 has been documented in fish and birds, however they were found neither in human nor in mouse (3). Surprisingly, an additional member, IRF11 was identified only in teleost fish (3). Three decades of research has allowed the determination of basic physiological function for each family member. In *Homo sapiens* IRFs are key mediators of signal transduction associated with host immune response, immunomodulation and hematopoietic differentiation. Accordingly, five functional subgroups can be distinguished: IRF1&2, IRF3&7, IRF4&8, IRF5&6, and IRF9 as a part of the Interferon stimulated gene factor 3 (ISGF3) complex. IRF1 and IRF2 promote the response of Th1 immune cells, whereas IRF3 and IRF7 are engaged in antibacterial and antiviral innate immunity. Expression of IRF4 and IRF8 is restricted to the lymphoid and myeloid lineages of the immune system (4), whereas they are crucial for B lymphocyte development and Th cell differentiation. In addition to a pro-inflammatory role, IRF5 is also involved in the regulation of apoptosis. Structurally similar IRF6 regulates proliferation and differentiation of keratinocytes (4). IRF9 together with members of the Signal transducers and activators of transcription (STAT) family, STAT1 and STAT2, forms the ISGF3 complex and transmits IFN type I and III induced signals (5). Based on a comparison of the C-terminal region of the IRF proteins, five members (IRF1, IRF3, IRF5, IRF7, and IRF9) were described as activators, whilst IRF2 and IRF8 as repressors. Furthermore, IRF2, IRF4, IRF5, IRF7, and IRF8 have been recognized as multifunctional agents, which both activate and repress gene transcription (6). In order to clarify the evolutionary relationship between IRFs we conducted phylogenetic analysis of IRF DNA binding domains (DBD). IRF-like proteins have been characterized in non-vertebrate deuterostomes, including the hemichordate—acorn worm, the echinoderm—sea urchin, the cephalochordate—lancelet and the urochordate—sea squirt (7). Based on our analysis vertebral IRFs can be divided into four subfamilies: IRF1 subfamily (including IRF1 and IRF2), IRF3 subfamily (including IRF3 and IRF7) IRF4 subfamily (including IRF4, IRF8, and IRF9) and IRF5 subfamily, which comprises of IRF5 and 6 (Figure 1A). This analysis is in agreement with previously published data on evolutionary conservation of the IRF family (7, 8).

All IRF family members are characterized by a multi-domain structure, which consists of: N-terminal DNA binding domain (DBD), a peptide Linker (LK) and IRF-association domain (IAD)1 or IAD2 within the C terminal activation domain (AD) (Figure 1B). A linker region connecting the DBD and IAD domains most likely folds into a domain rather than staying in extended form. A subset of IRF proteins (IRF3, 4, 5, and 7) contains an Auto-inhibitory region (AR) in their structure. This AR regulates their activity via different mechanisms involving conformational changes dependent or independent of phosphorylation events (9). Within the highly homologous DBD there are 5 precisely spaced tryptophan repeats forming the “helix-turn-helix” fold, essential for the recognition of similar DNA motifs with conserved GAAA repeats. The IFN regulatory element (IRE, NAANNGAAA) and the IFN-stimulated response element (ISRE, A/GNGAAANNGAAACT) are present in the regulatory regions of IFN-Is and IFN-stimulated genes (ISGs), respectively. IRF1 and IRF2 possess an approximately 177 amino acid long IAD2, while the rest of IRFs contain a conserved IAD1 (10). The more variable IAD is critical in mediating protein-protein interactions and thus defines the functionality of IRF family members.

As mentioned above, IRFs closely control transcriptional activation of IFN-Is and ISGs. As such they are crucial modulators of Toll-like receptor (TLR) and IFN signaling, key pathways of the innate immune system. Upon binding of Pathogen-associated molecular patterns (PAMPs) to the TLRs, or IFNs to the IFN receptors, a signaling cascade causes IRF activation and re-localization to the nucleus where they activate gene expression. IRFs exert the ability to interact with numerous transcriptional partners, including IRF family members, STATs as well as other co-acting transcription factors such as NF-κB (e.g., with IRF1 or IRF3) and PU.1 (with IRF4 and IRF8). These interactions allow IRFs to activate a broad spectrum of genes and control diverse transcriptional programs. Despite the clear similarity between IRF-DBD structures and the fact that they recognize the same consensus DNA-binding site, there are major differences in DNA binding affinities between family members. Moreover, depending on the binding partner, IRFs exhibit various DNA binding modes (Figure 1C). The ISRE binding site consists of two spaced GAAA elements, or ISRE half-sites, 2 or 3 bp apart. Activated IRFs might bind the ISRE as homo- and heterodimers. It has been reported that each of the IRFs forming a dimer bind the ISRE half-site on opposite sides of the DNA, in a proximal orientation (11) (Figure 1C). Based on the recently described crystal structure and binding models for STAT2 and IRF9 (12), we propose that within the ISGF3 complex, IRF9 interacts with one GAAA ISRE half-site, whereas the STAT1/STAT2 heterodimer via STAT1 binds the adjacent GAAA element spaced by 2bp (Figure 1C). An overview of the PU.1-IRF4/DNA complex provided by Escalante et al. revealed that PU.1 E26 transformation specific (ETS) and IRF4-DBD bind to a composite binding site formed on the opposite faces of the DNA in a head-to-tail orientation (Figure 1C). NF-κB, consisting of a p50/p65 heterodimer, specifically recognizes the NF-κB DNA element with the consensus sequence of GGGRNYYYCC (13), which is placed in such a way that IRF and NF-κB rest next to each other (or in close vicinity) on the DNA (Figure 1C). The DNA-binding specificity and affinity differences of these complexes collectively shape the transcriptional activity of IRFs.

Activation of IRFs is crucial in numerous essential signaling cascades. Thus, abnormalities of IRFs regulatory functions have been confirmed to play a key role in development of disease in all major areas, including acute and chronic inflammatory diseases, autoimmune diseases and multiple types of cancer. Accumulating evidence also suggests that different IRF dependent transcriptional mechanisms may be involved in the pathogenesis of these diseases. Participation of IRFs in divergent and overlapping molecular programs linked to their disease-specific functional role has motivated us to investigate IRFs as interesting therapeutic targets. Surprisingly, until now no direct inhibition strategies targeting IRFs have been reported. Known indirect IRF inhibitory strategies target IRF-dependent signaling at different levels, including inhibition of TLR or IFN receptors, IRF activators, IRF binding partners as well as blocking transcriptional or translational events. Nevertheless, none of these approaches proved to be effective enough to enter clinical trials. Over the years, structural models of IRF-DBDs and IRF-IADs have been systematically appearing in the PDB database. Available structures can be additionally divided into free cytoplasmic apo- and DNA bound nuclear holo-forms. Further investigating the architecture of IRFs, their possible interactions and IRF-mediated transcriptional regulatory mechanisms allowed us to propose a novel direct IRF-modulating strategy. This strategy employs our previously described pipeline approach Comparative Approach for Virtual Screening (CAVS) that combines comparative *in silico* docking to the IRF-DBD with *in vitro* validation of IRF inhibition (10).

With this review, we summarize the current knowledge of the different IRF-mediated transcriptional regulatory mechanisms and their role in health disorders. We postulate that specific target genes activated by the different IRF dependent transcriptional mechanisms have potential as promising novel disease markers. Going a step further, we hypothesize that the presented IRF-specific and pan-IRF inhibition strategies might represent the future for treating numerous immunological diseases. Hence, better understanding of IRF-dependent transcriptional programs and development of direct IRF inhibition approaches, could provide novel insight in the therapeutic, diagnostic and prognostic space occupied by IRFs.

## IRFs in the IFN and TLR Pathways

### IFN Signaling

IRFs are crucial modulators of production and IFN signaling. IFNs are a group of cytokines which regulate inflammation, cell proliferation, and apoptosis. IFNs are part of the first line of defense of the body against viral infections (14, 15). IFNs are divided into three subfamilies: Type I, Type II, and Type III IFNs. The Type I (IFN-I) subfamily consist of all subtypes of IFNα, and IFNβ, IFNκ, IFNω, IFNε, and signal via a receptor consisting of two subunits, interferon-alpha/beta receptor (IFNAR)−1 and IFNAR-2, which are expressed in nearly all cell types and tissues and are known to be paramount for a robust host response against viral infection (16). The Type II (IFN-II) subfamily consists of a single IFNγ (16) and acts via a receptor which consist of two interferon gamma receptor (IFNγR)-1 and two IFNγR-2 chains. IFN-II is mostly produced as a response to foreign antigens by T lymphocytes and natural killer cells. Finally, the third group of IFNs is the Type III (IFN-III) subfamily which uses the interferon lambda receptor (IFNLR) consisting of IL10R2 and IFNLR1 and is made up of IFNλ1, IFNλ2, IFNλ3 [reviewed in (17)] and the more recently discovered IFNλ4 (18). Like IFN-I, IFN-III possess potent antiviral activity (19).

**Figure 2:**
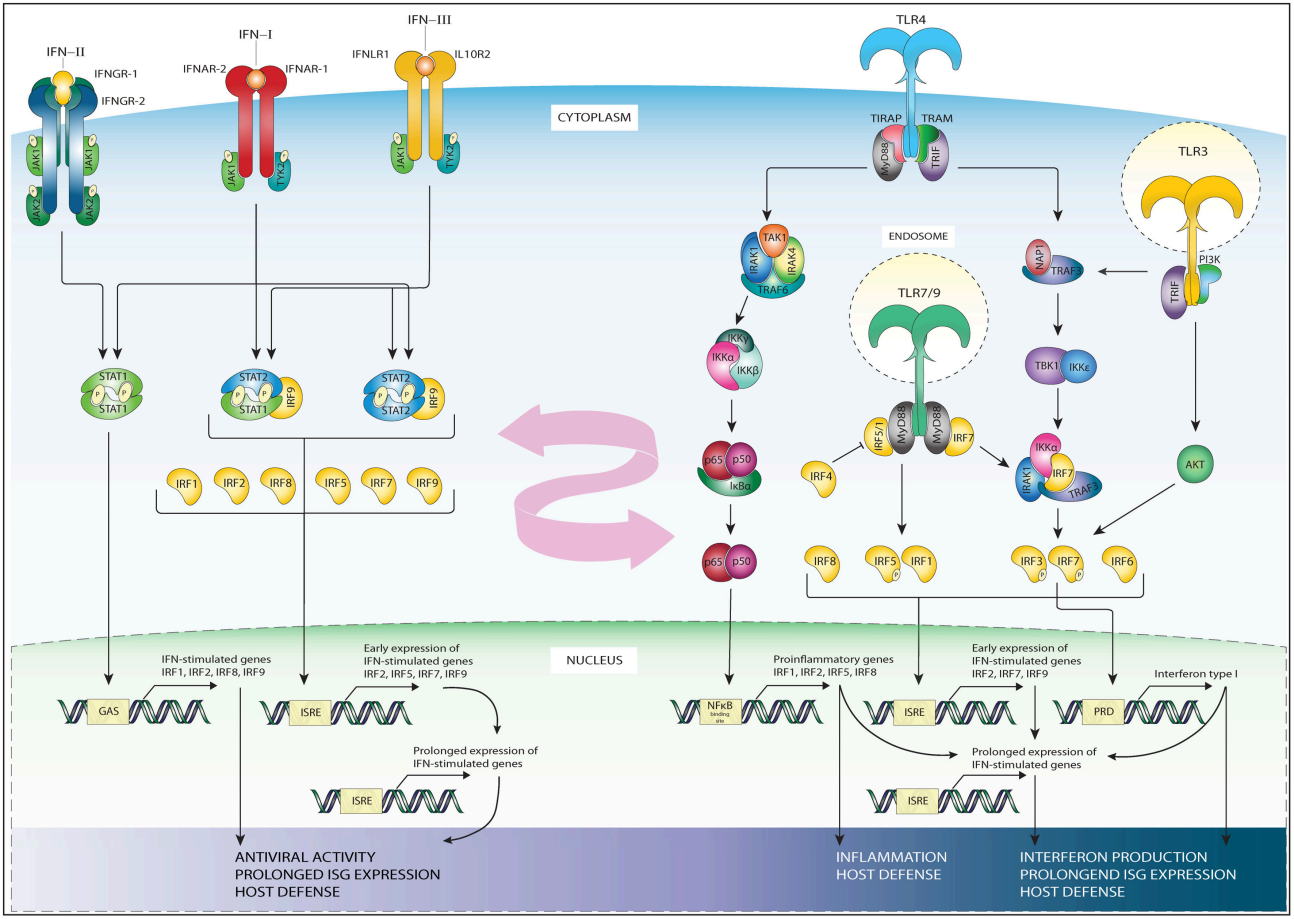
Schematic representation of IRFs in the TLR and IFN pathways. The three subfamilies of IFN signal through distinct receptors: IFN-II signals via a receptor which consist of two interferon gamma receptor (IFNγR)-1 and two IFNγR-2 chains (first left). IFN-I signal via the IFNAR receptor expressed in nearly all cell types and tissues (second left). IFN-III subfamily uses the interferon lambda receptor consisting of IL10R2 and IFNLR1 (third from left). While the signaling of IFNAR and IFN-lambda relies on phosphorylation of JAK1 and tyrosine kinase 2 TYK2, the interferon gamma receptor triggers STAT signaling via phosphorylation of JAK1 and JAK2. IFN-II specifically triggers STAT1 homodimer formation (most left), while IFN-I and IFN-III trigger ISGF3, (second from left), or STAT2/IRF9 in absence of ISGF3 (third of left). These complexes translocate to the nucleus to bind DNA on recognition sequences (GAS or ISRE, see bottom-left). The initial IFN stimulation leads to the early expression of ISGs and the transcription of IRF1/5/7/8/9 and STAT1, STAT2. The accumulation of newly synthesized transcription factors leads to a secondary, prolonged wave of ISG expression (bottom-left), contributing to antiviral activity and host defense. TLR4 signaling occurs through a MyD88-dependent (middle-right) and MyD88 independent (right) signaling cascade. In the MyD88 dependent signaling MyD88 recruits IRAK4 and IRAK1 leading to their phosphorylation, which in turn associates IRAK with TRAF6. TRAF6 activates TAK1, which in turn leads to phosphorylation of IKKα/β. The phosphorylation of these proteins results in their degradation and enables the translocation of NF-κB to the nucleus where it binds NF-κB binding sites (middle-right). The MyD88 independent signaling activates TRIF, which in turn via IKKε and TBK1 signaling phosphorylate IRF3 and IRF7 at their C-terminal serine/threonine cluster (right). Upon phosphorylation these IRFs translocate to the nucleus and bind ISRE or PRD sites on the DNA. TLR3 (most-right) also signals through TRAF and IRF3, or via the PI3K-Akt pathway. TLR7 and 9 signaling (right, down) goes via MyD88, TRAF, and IRF7, or via phosphorylation of IRF5. Down below in the figure the subsequent DNA recognition sites are listed, together with the general biological effects of gene activation, such as interferon production, prolonged ISG production and host defense. TLR3 and 4 signaling leads to upregulation of IFN beta, triggering the IFN-I pathway. This, together with the IRFs whose expression is upregulated by pathway activation (listed down below in the figure) provide cross-talk between the TLR and IFN pathways (pink arrow).

All types of IFN activate pathways based on Janus kinases (JAKs) and STAT signaling. While the signaling of IFNAR and IFNLR relies on juxta positioning and phosphorylation of JAK1 and tyrosine kinase 2 TYK2 (20), the IFNγR triggers STAT signaling via phosphorylation of JAK1 and JAK2 (21) (Figure 2). Subsequent JAK1 and TYK2-dependent phosphorylation of both receptor chains of IFNAR and IFNLR creates docking sites for STAT1 and STAT2 (22). Receptor bound STATs are activated via phosphorylation of tyrosine residue (Tyr)701 of STAT1 and Tyr690 of STAT2, which leads to heterodimerization and together with IRF9 to the formation of ISGF3. This heterotrimeric complex translocates to the nucleus and binds the ISRE sequence present in more than 300 ISGs, such as *ISG15, OAS1-3, IFIT1-3*, or *MX1* and *2*, which all are crucial in mediating antiviral activity (20). In a similar manner, but only in response to IFN-I, IRF9 and STAT2 homodimers can form an ISGF3-like complex (STAT2-IRF9) that can reinstate ISG expression in the absence of STAT1 (23–25).

The IFN-II pathway relies on the docking and phosphorylation of STAT1, but not STAT2. Therefore, IFN-II specifically triggers STAT1 homodimer formation known as IFN gamma activating factor (GAF). GAF translocates into the nucleus to activate genes containing the IFN gamma activating site (GAS) DNA element [consensus sequence: TTCN (2–4) GAA; (20, 22, 26)]. GAS binding of STAT1 also initiates IRF1 expression, resulting in the secondary expression of certain groups of ISGs (27, 28). Alternatively, the IFNγ-induced expression of the *CIITA, Gbp1*, and *Gp19* genes were shown to depend on both STAT1 and IRF1 (29–31) (Figure 2).

IFN-III signal via its distinct heterodimeric receptor to activate antiviral transcriptional responses largely overlapping with those of IFNAR in IFN-I signaling. However, were IFNα receptors are expressed on nearly all cell types, IFNλ receptors are mainly restricted to cell types of epithelial origin (19) (Figure 2, left side).

The initial IFN-I, IFN-II and IFN-III stimulation leads to the transcription of IRF1, IRF5, IRF7, IRF8 and even STAT1, STAT2, and IRF9 themselves (4, 6, 26). As IRFs bind a specific GAAA motif (IRF element; IRE), IRFs can bind positive regulatory domains (PRD-I) containing such IREs as well as ISREs. IREs are not recognized by ISGF3. Moreover, the accumulation of newly synthesized STAT1, STAT2 and IRF9 proteins in the cytoplasm can lead to the creation of new transcription factors in an unphosphorylated form. When the amount of phospho-proteins subsides, these unphosphorylated complexes such as unphosphorylated ISGF3, unphosphorylated STAT1 dimer or STAT2/IRF9 complex can support or take over the role of phosphorylated complexes in sustaining the expression of ISGs [reviewed in (26)]. Together, this feedforward loop controls the prolonged expression of many ISGs instrumental in generating a potent antiviral response and host defense.

### TLR Signaling

As mentioned above IRFs are also instrumental in the action of TLRs. As part of the innate immune system, TLRs are one of the earliest surveillance systems and line of defense against primary infections by pathogens (32, 33). Currently 10 distinct TLRs have been identified in humans. These TLRs recognize a wide range of PAMPs (e.g., Bacterial lipopolysaccharides) and tissue damage associated molecular patterns (DAMPs; e.g., Heat-shock proteins, uric acid and ATP). In response to these PAMPs or DAMPs, TLRs initiate an inflammatory signaling cascade which in most cells leads to a swift and potent upregulation of inflammatory gene expression. These inflammatory genes include endothelial adhesion molecules, chemokines, and inflammatory cytokines among others (34).

From all TLRs, the most well-known is TLR4, that recognizes lipopolysaccharide (LPS), a component of many bacteria. Besides LPS, other TLR4 ligands include several viral proteins and a variety of endogenous proteins such as low-density lipoprotein, beta-defensins, and heat shock proteins (35). TLR4 is activated upon ligand binding. As such TLR4 downstream signaling pathways either work in a manner dependent on the universal adapter protein called Myeloid differentiation primary response 88 (MyD88), or in a MyD88-independent way (Figure 2, right side). In the MyD88-dependent arm of TLR4 signaling, MyD88 recruits IL-1R-associated kinase 4 (IRAK4), and IRAK1 which leads to their phosphorylation and in turn results in the association of IRAK with the ubiquitin ligase Tumor necrosis factor (TNF) receptor-associated factor 6 (TRAF6). TRAF6 then activates transforming growth factor-β activating kinase (TAK1), which subsequently leads to phosphorylation of IκB protein complex, composed of the kinases IKKα, IKKβ, and IKKγ. Normally IκB sequesters the p50-p65 heterodimer NF-κB in an inactive form in the cytosol (36). However, the phosphorylation of the IκB proteins results in their degradation and this enables the translocation of NF-κB to the nucleus (37). NF-κB is a transcription and signaling protein complex of proteins that regulates cytokine production and cell survival (38). All NF-κB family members contain a Rel homology domain in their N terminus, allowing for the formation of multi-protein DNA-bound complexes (39). Upon NF-κB nuclear translocation it can induce expression of pro-inflammatory cytokine genes, such as TNFα, IL-6, and IL-12p40, crucial for the generation of the acute phase response, and the differentiation of neutrophills and natural killer cells (40, 41). Moreover, NF-κB also binds to the promoters of *IRF1, IRF2, IRF5*, and *IRF8*, upregulating their expression, providing for the activation of ISGs and so forming a link between the TLR and IFN pathways (42).

The MyD88-independent pathway was initially postulated based on studies revealing that both TLR3 and TLR4 ligands are still able to upregulate the expression of IFN-I and IFN inducible genes in mice deficient for MyD88 (43, 44). In this context, TLR3 and TLR4 activate the adapter protein TIR-domain-containing adapter-inducing interferon-β (TRIF), which in turn via IKK kinase signaling activate IRF3 (45). IKK kinases IKK-1 and TANK-binding kinase 1 (TBK1) phosphorylate IRF3 and IRF7 at their C-terminal serine/threonine cluster [(46); see further down below]. IRF3 is constitutively expressed and resides in the cytoplasm, but gets internalized to the nucleus upon phosphorylation. Here, IRF3 will initiate the transcription of *IFN*β, which in an autocrine fashion through the IFNAR complex further stimulates ISGF3-dependent ISG expression (Figure 2; middle part) (26). Endocytosis of the TLR4 complex has been suggested to play a role in determining the order in which the MyD88 dependent and independent pathways are induced, with the first being activated at the plasma membrane, and the latter from early endosomes (47).

Other TLR pathways in which IRFs play a role are TLR3, 7, and 9. TLR3 recognizes double-stranded RNA and therefore is crucial in the host response against viral infection (43). An example of a double-stranded RNA virus is the Reoviridae, a common cause of gastroentritis in children (48). TLR3 mediates TRIF induced phosphorylation of IRF3, similar to the MyD88 independent signaling in TLR4. Moreover, TLR3 can also interact with PI3K and phosphorylate Akt, which leads to further activation of IRF3 and IRF7 [reviewed in (33)]. IRF6 has recently been revealed to play a role in TLR3 signaling in keratinocytes (49). TLR3 stimulation in these epithelial cells enhanced the expression of IFNβ, IL-23p19, IL-8, and CCL5. Silencing of IRF6 resulted in an even higher expression of IFNβ, but a decrease in IL-23p19 (49).

TLR7 and TLR9 are strongly expressed in plasmacytoid dendritic cells (pDCs), where they are responsible for a high level of IFN-I expression in response to viral infection (50, 51). TLR7 recognizes single stranded RNA, and is thus of great importance in host defense against viral infection with HIV or Hepatitis C virus (HCV) (52, 53). TLR9 is a receptor for unmethylated CpG DNA, commonly found in bacteria (54). Both these TLRs rely solely on MyD88 for their downstream signaling. Protein kinases from the IRAK family are important for the MyD88—IRF interactions and subsequent activation. MyD88, TRAF6, and IRAK4 form a complex in the endosomal vesicles of pDCs (55), and there interact with IRF7. IRF7 is constitutively present in the cytoplasm, but upon phosphorylation moves to nucleus and activates the expression of different *IFN*α subspecies (56). In an effort to elucidate the underlying mechanisms of MyD88-mediated TLR signaling, Takaoka et al. discovered a role for IRF5 in this process (57). The IRF5 deficient mice used in this study showed an impaired induction of inflammatory cytokines such as IL-6, IL-12, and TNF after stimulation with several TLR ligands. Other studies found IRF5 to be induced by TLR7 signaling (58). Using *in vivo* reporter assays, Schoenemeyer et al. demonstrated that TLR7 activates IRF5 and IRF7 but not IRF3. They further demonstrated via IRF5 knockdown that TLR7 signaling through IRF7 requires IRF5 to activate IFN-Is (58). Indeed, in 2013 Yasuda et al. confirmed IRF5 importance by demonstrating that this IRF was required for TLR7 and TLR9 induced pro-inflammatory cytokine IL-6 and IFNα/β production in dendritic cells (59). Together, the conclusions of these studies implicate IRF5 and IRF7 as critical mediators of TLR7 and TLR9 signaling.

Signaling through the TLR pathways induces transcription of IFNα, IFNβ, and IRF1,2,5,7,8, and 9 (Figure 2). The promoters of *IFN*α and *IFN*β genes have IRE containing PRD-I, making them susceptible to IRF-induced upregulation (1, 2). Moreover, NF-κB also binds a PRD site in the promoters of *IFN*β and further enhances transcription of this gene (60). Indeed, in the *IFN*β promoter, PRD-I or PRD-IV bind IRF3 and 7, PRD-II NF-κB, and PRD-IV binds ATF-2/c-Jun, which all together form the *IFN*β enhanceosome that has been proven to be an essential component for virus-induced *IFN*β transcription (60–62). This increased IFNα/β production will trigger the subsequent IFN-I signaling pathway to upregulate ISG expression and so further enhance the host immune response. Indeed, many ISGs exhibit binding sites not only for IRFs and STATs, but also for NF-κB, mediating their cooperation in response to TLR and IFN signaling (28, 63). Moreover, the IRFs produced in the initial wave of TLR signaling further fortify the expression of ISGs and so facilitate prolonged pathway activation and expression during inflammation. The fact that most of the IRFs used in both TLR and IFN pathways overlap, allows for further cross-talk, synergy, and signal integration between these pathways, which is an important aspect of the host defense against pathogens (Figure 2).

Although this review focuses on TLR and IFN-mediated IRF activation, Retinoic acid inducible gene I (RIG-I)-like receptors (RLRs) also utilize IRFs to induce the expression of cytokines, or exert gene expression independent effects. These pattern recognition receptors are also of great importance for antiviral responses and are addressed in more detail elsewhere (64–67).

## IRF Dimers in DNA Binding and Transcriptional Activation

IRFs activated in the TLR and IFN signaling pathways bind the ISRE as homo- and heterodimers, in which each IRF contact the ISRE half-site on opposite sides of the DNA, in a proximal orientation (11) (Figure 1C). A deeper understanding of dimerization of IRFs and DNA binding, comes from the analysis of structural data provided in the literature by means of X-ray crystallography or NMR. Crystal structures of IRF1, 2, 3, and 7 have been used to describe their DNA binding modes. The crystal structure of the *Mus musculus* IRF1-DBD in complex with a 13nt DNA fragment from a PRD1 element containing GAAA core sequence was solved in 1998 (PDB Id 1IF1). Topologically the IRF1 DNA-binding region is similar to a helix-turn-helix DNA-binding domain and includes a four-stranded antiparallel β-sheet and three large loops (L1-L3) connecting the different secondary structure elements, but its mode of DNA interaction is distinct. Thus, four amino acids mediate contact with DNA in the major groove (Arg82, Cys83, Asn86, and Ser87). Additionally, three tryptophan residues (Trp11, Trp38, and Trp58) are involved in hydrogen bonds and van der Waals contacts with the sugar-phosphate backbone (68). The IRF2-DBD-DNA complex reveals a very similar spatial structure, that could be explained by 80% sequence identity with IRF1 within the first 113aa, responsible for DNA binding. This structural similarity results in very similar binding affinities for both proteins (69, 70).

To date, the crystal structures for the majority of the IRF-DBDs were deposited in the PDB, including IRF3- (*Mus musculus*–3QU6), IRF4- (*Homo sapiens*–2DLL), and IRF7-DBDs (*Mus musculus*–3QU3). Despite the significant similarity between DBD structures of different IRFs and the fact that they all recognize the same consensus DNA binding site, there are major differences in DNA binding affinities between family members. Analysis of the DBDs from IRF3 and IRF7 reveals that this phenomenon can be explained by differences in flexibility and conformational changes in the loops, in particular L1. In IRF3 this loop is disordered in the apo-form and becomes ordered, when DNA is contacted. In contrast to IRF3, IRF7 L1 is ordered and stabilized by two hydrophobic residues (Phe45 and Leu50) that fold back into the core of the protein in the apo-form and during DNA-binding a 2Å rigid body transition is observed (71). Taken together, variable intrinsic loop flexibility of IRFs may determine their binding specificity and differences in binding affinities.

**Figure 3:**
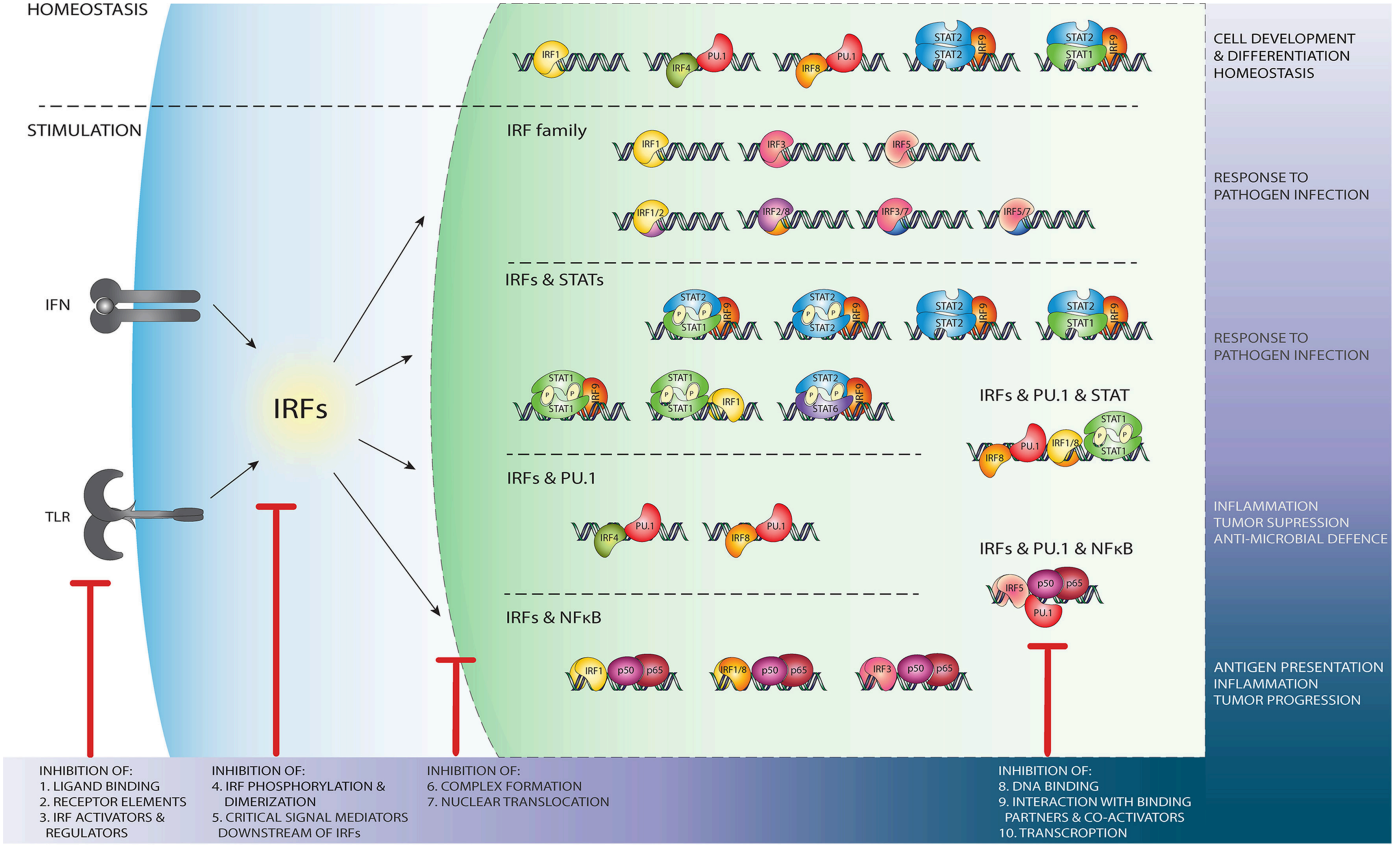
Schematic representation of the wide variety of IRF-mediated transcriptional regulatory mechanisms and their function. Independent of TLR and IFN stimulation, PU.1/IRF binding regulates leukocyte development and differentiation, while IRF1 homodimers, u-ISGF3 and u-STAT2/IRF9 maintain ISG expression in homeostasis (upper panel). After stimulation of the TLR and IFN pathways, DNA binding of IRF homo- and heterodimers (second panel from above), IRF/STAT complexes (middle panel), IRF/PU.1 complexes (second panel from bottom), or NF-κB/IRF (bottom panel) dependent mechanisms initiate or enhance ISG transcription. Potential IRF inhibition strategies. Red sticks indicate several points at which IRFs activity might be blocked by targeting: (1) ligand binding to the receptor e.g., TLR; (2) active components of the receptors, such as Jak2; (3) important IRF regulators and activators, such as NF-κB; (4) events such as phosphorylation, homo- and hetero- dimers formation; (5) critical mediators downstream of IRFs; (6) complex formation with other TF such as STATs; (7) IRFs ability to translocate to the nucleus. IRFs activity might be also modulated by preventing DNA binding, either directly (8) or by blocking interaction with binding partners (9) such as PU.1 or NF-κB. IRFs transcription (10) can be disrupted by RNAi and ncRNA mechanisms.

IRF3 is known to form homodimers upon viral infection [reviewed in (72, 73)]. Crystal structures of the IRF3 transactivation domain reveal a unique auto-inhibitory mechanism. As such the auto-inhibitory elements surrounding the IAD, in a closed condensed form, create a hydrophobic core that maintains the protein in an inactive state. Release of the hydrophobic active site upon phosphorylation leads to a conformational change, unveils the DBD and enables DNA binding (74, 75). Moreover, phosphorylation-dependent IRF3 dimerization results in a unique acidic pocket formation, serving as a binding site for other transcription factors such as CREB-binding protein (CBP)/p300 (75, 76). Transcriptional activity of IRF3 is controlled by phosphorylation events on Ser385 or Ser386 induced by viruses and/or dsRNA (46, 76, 77). Additionally, phosphorylation mediated by the IKK related kinases, targets the C-terminal serine/threonine cluster between aa 396-405 (46, 77). This IRF3 homodimer is seen as the master and primary transcription activator of *IFN*β and *IFN*α*4* genes, leading to the activation of the IFN-I pathway and subsequent ISG expression [reviewed in (72)]. The proposed model of transcriptional activation of IRF5 and IRF7, similar to IRF3, involves conformational changes induced by C-terminal phosphorylation followed by homo- and heterodimerization and translocation to the nucleus. However, it seems that other IRF family members may work through different activation systems independent of phosphorylation. For example IRF4, which is characterized by low affinity DNA binding, possesses an AR covering the last 30 amino acids of the IAD. In an auto-inhibitory mechanism model proposed by Remesh et al., it was suggested that the AR directly interacts with the DBD and leaves the protein in an auto-inhibited, inactive state. Upon interaction with a binding partner, the protein structure is reorganized, unmasking the DBD and allowing IRF4 to contact DNA. The same group presented a structural characterization of full-length IRF4 based on SAXS (small angle X-ray scattering) studies, which revealed that the flexible linker between DBD and IAD forms rather a domain-like structure that maintains in an extended form. Moreover, it may play a crucial role in regulation of IRF4 function. Due to the high structural similarity, it can be speculated that the regulation of IRF8 activity proceeds in a comparable manner (9, 78) (Figure 3).

A genome-wide study in which protein-binding microarrays were used to characterize the DNA binding of IRF3/5/7 homodimers revealed that besides common binding sites, a large number of dimer-specific DNA binding sites are present in the human genome. This suggests that dimer specific binding can result in dimer-specific gene regulation (11). Similar to homodimers, IRF heterodimers form a complex with one IRF on each side of the DNA helix, both contacting the full length ISRE sequence. Both IRF5 and IRF7 are expressed constitutively in monocytes, B lymphocytes, and precursors of dendritic cells (DCs). When IRF7 gets phosphorylated, it can interact with IRF5 and form a heterodimer (Figure 3). Through mapping of the interaction domain, Barnes et al. showed with the use of fibrosarcoma and lymphoma cells that IRF5/IRF7 heterodimers are formed through the amino terminus and masks the DNA binding domain, resulting in an alteration in the enhanceosome complex of IFNα activated gene sets (79). In this way, the IRF5 and IRF7 heterodimer can play a critical role in the induction of IFNα genes in infected cells. Genes expressed after IRF5/7 heterodimer initiation were not only encoding inflammatory and antiviral proteins, but also pro-apoptotic proteins and proteins of other functional categories (80, 81). IRF1 and IRF2 are also known to form a heterodimer which has been shown to regulate transcription of the Epstein-Barr virus EBNA1 gene in infected fibroblasts (82). Moreover, IRF1 and IRF2 are both bound to and regulate *Cox-2* an prostaglandin E2 genes upon IFNγ or LPS stimulation (83). Chromatin immunoprecipitation and RNA sequencing studies of genes bound and activated by IRF8, IRF1, PU.1, and STAT1, revealed the existence of an IRF1/IRF8 regulome, which plays critical roles in inflammatory and antimicrobial defense, such as neuroinflammation and tuberculosis (84). Furthermore, the expression of IL-1β in IRF8-expressing reactive microglia in Peripheral nerve injury is dependent on IRF1 further suggesting the existence of an IRF1/8 regulome (85).

The most recognized IRF heterodimer is IRF3/IRF7. At specific stages during inflammation, IRF3 and IRF7 physically interact. In human fibroblast cell lines viral infection activated IRF7 and consequently upregulated MAP3K8, a kinase inhibiting IRF3 dimer formation and promoting the formation of IRF3-IRF7 heterodimers (86). These heterodimers were necessary for limiting viral replication *in vitro* (86).

## IRFs in Complex Formation with other Transcriptional Regulators

IRFs exert the ability to interact with numerous transcriptional partners, not only within the IRF family but also outside with STATs as well as other co-acting transcriptional regulators such as PU.1. These interactions allow IRFs to activate a broad spectrum of genes and control diverse transcriptional programs.

### STATs

The ISGF3 complex (assembly of IRF9, STAT1 and STAT2), recognizing the ISRE element, is an example of a cooperativity between IRF and STAT transcription factors. The direct interaction between the STAT2 coiled-coil domain (STAT2-CCD) and the IRF9-IAD is critical for the function of ISGF3 and the antiviral response. Studies by Rengachari et al. showed that the overall architecture of these domains is similar to that of other STATs and IRFs (12). Crystal structures of the STAT2-CCD/IRF9-IAD complex (*Mus musculus*–5OEN) solved by Panne‘s group revealed several important adaptations that explain the selective interaction between STAT2 and IRF9. Indeed, the IRF9-IAD is missing the regulatory apparatus that is used for IRF auto-inhibition in the latent form, and in the activated state enables IRF dimerization and interaction with the transcriptional co-activators CBP/p300. Accordingly, IRF9 interacts with the tip of the STAT2-CCD using the convex surface of the β-sandwich core of the IAD domain. While the same surface is available in other IRFs, amino acid substitutions at the key anchoring points account for the preferential IRF9-STAT2 interaction. Taken together, these adaptations explain why IRF9 binds constitutively and selectively to STAT2 and demonstrate that the observed interface is required for ISGF3 function in many cells (Figures 1C, 3).

Together with others, our group described the existence of an ISGF3-like complex of IRF9 and STAT2 (STAT2/IRF9), which in absence of STAT1 restores IFN-I responses. STAT2/IRF9, like ISGF3 also recognizes the ISRE sequence (23–25) and upregulates a similar subset of IFN-stimulated genes as compared to ISGF3 in STAT1 deficient cells (87). However, the genes which were activated by both ISGF3 and the STAT2/IRF9 complexes did differ in expression profiles: ISGF3 driven activation appeared to be early and transient, while STAT2/IRF9 gave rise to a delayed but prolonged activation profile (26, 87, 88). A STAT2/STAT6/IRF9 protein complex has also been described. It was found specifically in B-lymphocytes after IFNα stimulation. In these cells, IFN stimulation lead to the activation of STAT6 and the formation of STAT2/STAT6/IRF9 complexes, which may allow for cell-specific modulation of gene expression (87, 89). Furthermore, the existence of another IFN-responsive protein, ISGF2 was hypothesized (90). However, this later was shown to be IRF1 (91). STAT1 homodimers are also known to form transcription complexes together with IRF1. Genome-wide studies to the extent of STAT1 and IRF1 cooperation in HeLa cells showed that co-binding of STAT1 and IRF1 to proximal or distal ISRE and GAS motifs occurs twice as often as STAT1 alone, and even 6 times more in the *MHC I* locus, crucial for antigen presentation. Also, dual binding of IRF1 and STAT1 vs. single binding of IRF1 distinguished IFNγ induced ISGs vs. cell-specific IFNγ resistant ISGs (92).

In an unstimulated state, some ISG expression is present and known to be crucial for controlling cellular susceptibility to viral infection (93). Under these conditions, unphosphorylated (U-)ISGF3, but also U-STAT1 and U-STAT2/IRF9 are proposed to mediate constitutive IFN-independent expression of ISGs to protect against viral infection [reviewed in (26); Figure 3, upper panel]. Our group has shown that U-STAT2/IRF9 increases basal expression of several ISGs including IFN-induced apoptosis mediator IFI27, activator of viral RNA degradation OAS2, double-stranded RNA binding protein OASL, and the Hepatitis C associated IFI44 in STAT1-knockout (KO) cells overexpressing STAT2 and IRF9 (87). Furthermore, basal DNA-binding of U-STAT1 in combination with IRF1 is connected to the constitutive expression of some targets, including the Proteasome subunit LMP2 and cellular membrane transporter TAP2, to maintain their constitutive expression (94).

### PU.1

The transcription factor PU.1 (also known as Spi-1) is a protein of the ETS family, and has an ETS domain with which it can bind DNA at a sequence known as the PU box [a core RGAA DNA motif situated within a longer sequence; (95, 96)]. PU.1 is expressed in leukocytes such as macrophages, osteoclasts, neutrophils, and B-lymphocytes (97). Gene activation through IRF & PU.1 happens during homeostasis or is upregulated by transcription factors such as Nrf2 (98) (Figure 3). Due to a characteristic low DNA binding affinity and presence of an AR, IRF4 requires interaction with different binding partners, such as PU.1, to effectively bind DNA (99). Escalante et al. reported the structure of a ternary complex formed with the DNA binding domains of PU.1 and IRF4 on a composite DNA element (structure not available in RCSB PDB). The DNA contacted by this complex contorts into an unusual S shape that juxtaposes PU.1 and IRF4 for selective electrostatic and hydrophobic interactions across the central minor groove (99). Considering that PU.1 recruits IRF4 or IRF8 to DNA, and exhibits an anti-cooperative interaction with IRF1 and IRF2, structural characteristics of the IRF4-PU.1 complex with DNA provides insight into the structural basis of cooperativity and anti-cooperativity between ETS and IRF factors. The proposed IRF4 auto-inhibitory strategy suggests that the AR directly interacts with the DBD and leaves the protein in an auto-inhibited, inactive state. Upon interaction with a binding partner, the protein structure is reorganized, unmasking the DBD and allowing IRF4 to contact DNA. Due to the high structural similarity, it can be speculated that the regulation of IRF8 activity proceeds in a comparable manner (9, 78). These complexes can be formed independently of the TLR and IFN activated pathways, as IRF4/PU.1 and IRF8/PU.1 are crucial for leukocyte development (100). However, PU.1 can also be phosphorylated and activated on Ser148 in its PEST region by LPS treatment (101) (Figure 3), and by the IFN-pathway signaling protein JAK2 (102), JNK1 (103), as well as IFNα (104). The corresponding IRF4/PU.1 and IRF8/PU.1 co-activating complexes recognize and bind to the PU.1/ISRE binding motif, a variation of the classical ISRE sequence, which has a 5′RRRGAAGT-GAAANY 3′ consensus motif (105–107). Indeed, IRF4 and IRF8 were found to co-operate with PU.1 to activate specific inflammatory genes such as CD20, Ig light chain enhancers, IL-18 and IL-1β (105–107). PU.1 binds the PU.1/ISRE binding motif in gene promoters or enhancer regions, and then recruits IRF4 or IRF8 which interact with PU.1 on a phosphorylated PEST domain, a part of the PU.1 peptide sequence rich in proline, glutamic acid, serine, and threonine (105, 108). The PU.1-IRF4 dimer can potently represses the expression of the immunoglobulin lambda gene (the small polypeptide subunit of any antibody), and thus is of critical importance in the regulation of B cell gene expression (108). On the other hand, PU.1/IRF8 activity is necessary for the regulation of the macrophage expressed cytokine IL-18 (109). Mancino et al. demonstrated a distinct difference in gene regulation by basal IRF8-PU.1 compared to LPS induced complexes. Basal IRF8-PU.1 binding upregulated a broad panel of genes essential for macrophage functions, while after LPS stimulation increased IRF8 expression together with other IRFs or AP-1 family members could activate other genes not premarked by PU.1 (110). Both IRF4-PU.1 and IRF8-PU.1 are able to bind to a PU.1-IRF composite element in the promoter of *IL-1*β. However, when IRF1 or IRF2 were co-expressed with IRF4-PU.1 or IRF8-PU.1, the *IL-1*β promoter activity was increased over 100-fold as compared to that observed in cells with IRF4-PU.1 or IRF8-PU.1 alone (111). These studies provide evidence for an enhancing role of IRF co-activating complexes. A more in-depth study of PU.1-IRF dependent transcriptional mechanisms is presented in the review of Marecki et al. (111) (Figure 3).

### CREB & BATF

CREB is a transcription factor which recognizes and bind cAMP response elements (CRE, consensus sequenc e 5'-TGACGTCA-3′) on the DNA (112, 113). After CRE binding, CREB needs to be co-activated by CBP before gene activation can commence (114). Both CBP and p300 exert histone acetyltransferase activity, allowing for the stabilization and interaction of additional proteins with the transcription complex (115). CBP and p300 are paralogs and thus highly similar in build-up (116). CREB and CBP/p300 were found to have an important co-activating role in IFNβ regulation. They do so via the recognition element PRD-IV [sequence TGACGTC/A A/G; (117)]. Binding of ATF-2 or CREB-1 proteins to this element was found to be required for virus induced IFNβ expression (117). IRF1 and CREB also form an activating complex upon stimulation with leptin, which can bind the distal promoter of trombosponin-1 and activate the transcription of this gene (118). High level of this gene is associated with vascular injury, diabetes and atherosclerosis (118).

In macrophage and DC differentiation, in which IRF4 and IRF8 are known to play crucial regulatory roles. A chromatin immunoprecipitation study revealed that IRF4 together with The Basic leucine zipper transcription factor (BATF) bound DNA in close proximity of DNA sequences that recognize AP-1 family members (119, 120). BATFs are proteins belonging to the larger AP-1/ATF superfamily of transcription factors, able to dimerize with proteins from the Jun family (121). When B-Jun and BATF form a dimer, they are able to bind DNA on an AICE motif (5'-TGAnTCA/GAAA-3'), and subsequently recruit IRF4 or IRF8 to this site to initiate promoter activation (119, 120). Indeed, knockout studies have shown that BATF binding was diminished in IRF4 deficient T lymphocytes, and IRF4 binding was diminished in BATF deficient T lymphocytes (122). In this manner, BATF-IRF4 and BATF-IRF8 complexes can regulate a narrow set of genes necessary for leukocyte differentiation in a specific manner. BATF2, one of the lesser known BATF family members has been shown to play roles in T-lymphocyte, B-lymphocyte, and DC differentiation (123), and was shown to be highly expressed in IFNγ stimulated M1 type macrophages, contrary to M2 type macrophages (124). Furthermore, BATF2 regulated genes were demonstrated to be enriched with IRF1 binding motifs, while co-immunoprecipitation studies showed an association between BATF2 and IRF1 (124).

## IRFs in Co-Binding Mechanisms of Transcriptional Regulation

Another layer of transcriptional regulation in which IRFs play a role can be found in enhancing and co-binding mechanisms. Transcription factors including IRF3/IRF7, ATF-2/c-Jun, NF-κB and architectural protein HMGI(Y) assemble together to form an enhanceosome (62, 77). Cooperative binding of transcription factors to the IFNβ enhancer region stimulates transcription of the *IFN*β gene. It has been observed that binding-induced changes in DNA conformation and not the surface of protein-protein interactions is crucial for cooperative binding and transcriptional activation. Detailed analysis of this enhanceosome assembly was conducted on crystal structures of the DNA-binding domains of human IRF3, IRF7, and NF-κB bound to the *IFN*β enhancer (PDB IDs–1T2K, 2O61, 2O6G) (62, 125). Additionally, IRF3 has been shown to interact with CBP, STING, MAVS, and TRIF adaptor proteins. Studies on the structure of the IRF3 phosphomimetic mutant S386/396E bound to CBP (5JEM) suggested that a conserved *p*L*x*IS motif is responsible for this cooperation.

A wide range of studies have identified a plethora of genes which are upregulated by the co-activating effects of NF-κB and IRFs. The first suggestion of such co-activating effects was of IRF1 and NF-κB, present within the IFN regulatory element (IRE) of the *IFN*β promoter. NF-κB upregulates IFNβ gene expression by binding two recognition sites in its promoter. These recognition sites flank the PRD-I motif on which IRF1 binds (1, 126, 127). IRF1/NF-κB co-activation therefore relies on both ISRE and κB binding, in which IRFs and NfκB sit next to each other on the DNA (Figures 1C, 3). IRF1 by itself is enough to upregulate IFNβ after Newcastle Disease viral infection, while NF-κB alone was shown not to induce upregulation. However, as mentioned before, the upregulation of IFNβ was far more potent when IRF1 and NF-κB bound simultaneously to its promoter region (1).

Cross-regulation between NF-κB and IRF3-activated signaling pathways is also evidenced by the presence of multiple κB and ISRE binding sites in gene regulatory regions (42). The mechanism of IRF3/NF-κB is the same as described for IRF1/NF-κB. Concerted action of NF-κB and IRF3 is mandatory for transcriptional activation of multiple genes, including chemokines *Cxcl10* and *Ccl5*, activator of inflammasome *Gbp5*, Immune-Responsive Gene 1, and *IFN*β*1*. Detailed activation kinetics analysis suggested that individual genes within this small cluster use distinct regulatory mechanisms (128, 129). Moreover, virus-induced genome-wide occupancy of IRF3 and p65/RelA binding sites correlated with co-binding of other antiviral transcription factors (130). Mechanistically, NF-κB was found in a genome-wide study of Wienerroither et al. to recruit the mediator kinase module of the transcription complex, while STATs in ISGF3 contact the core mediator module of the transcription complex, both necessary for successful gene transcription (131). Indeed, other genome-wide studies established that also in genes activated by IRF3 and RelA binding, MED1 and Polymerase II binding occurred at overlapping positions in the promoters, suggesting their roles in transcription complex recruitment (130).

More recently, interplay between IRF5 and NF-κB has also been revealed. The induction of the TLR7 pathway by Imiquimod lead to the upregulation of IRF5 via the activation of NF-κB and PU.1, which were found to bind to the first two exons of the IRF5 gene (132). Moreover, NF-κB plays a role in the recruitment of IRF5 to the non-canonical composite PU.1-ISRE binding sites in promoters of inflammatory genes in macrophages after LPS stimulation (133).

Together, these studies suggest that IRFs collaborate globally with NF-κB and other co-activators utilizing diverse regulatory mechanisms to precisely induce distinct transcriptional regulatory networks.

## IRFs in Inflammation, Immunological Disorders and Cancer

TLR and IFN signaling cascades are well-ordered processes, regulated by multiple transcription factors, including IRFs. As a consequence, impaired activity of IRFs and the resulting aberrant ISG expression is implicated in a broad range of inflammatory and immunological diseases and cancer.

### IRF1 & 2

IRF1 is implicated in many diseases. Extensive studies have been carried out concentrating on the role of this IRF in viral and bacterial infections. For example, polymorphisms in the *Irf1* gene are reliable indicators for susceptibility to the development of chronic hepatitis B and C (134). Moreover, IRF1 has been implicated in the development of gastritis and atrophy in *Helicobacter pylori*-infected wild type (WT) mice (135). IRF1 DNA binding was also enhanced in macrophages *ex vivo* infected with *Mycobacterium tuberculosis*, and IRF1 mRNA expression was elevated in bronchoalveolair lavage samples of tuberculosis patients compared to samples of healthy volunteers (136). Furthermore, mice lacking IRF1 which were infected with *M. tuberculosis* displayed a diminished level of pulmonary inducible NO synthase (iNOS) mRNA expression and significantly increased CD4/CD8 ratio as compared to WT mice (137, 138). Moreover, IRF1 activity has been implicated in the expression of classic and non-classic MHC class I and MHC class II genes and subsequent development of thymic CD8+ T-cells. Thus, implying a role for IRF1 in antigen presentation (139, 140).

IRF1 is also connected to a variety of cancers. IRF1 KO mice studies provided proof for IRF1 antitumor functions (141, 142) (Figure 3). IRF1 KO mouse embryonic fibroblasts (MEFs) are more susceptible to oncogene-induced cell transformation (143). Moreover, they do not undergo cell cycle arrest in response to DNA damage (141). *IRF2* originally identified as an IRF1 antagonist acts as an oncogene, promoting cellular transformation. Its role in suppression of IFN-I signals has also been well-documented (144). Moreover, IRF2 was found to repress NF-κB induced *MHC-I* gene expression, involving more IRF family members in the low-MHC-I mediated Neuroblastoma disease progression (145). Another target of IRF1 and IRF2 that is implicated in neuroblastoma is Caspase-8 and its family member Caspase-7. Caspase-7 & 8 are involved in the early stages of apoptosis signaling by death receptors, and silencing this genes have been proposed to play a crucial role in tumor progression (146–148). Indeed, the restoration of Caspase 8 expression sensitized Neuroblastoma cells to death receptor signaling and cytotoxic drugs (149).

Our group and others, have studied to the role of STATs and IRFs in atherosclerosis [reviewed in (10, 63, 150)]. IRF1 is an important regulatory factor in the protection against vessel wall damage. Mice deficient in IRF1 were highly susceptible to neointima formation after vessel injury. IRF1 phosphorylation correlated with cell cycle arrest in coronary artery smooth muscle cells (151). Moreover, IRF1 induced nitric oxide production, which is known to attenuate endothelial dysfunction (152). Finally, increased expression of IRF1 mediates the endogenous IFNγ-promoted intimal thickening in immune-deficient Rag-1 KO mice after vascular injury (152, 153). STAT1 has also been identified as an important regulator of foam-cell formation and atherosclerotic lesion development in mice models (154). Increased STAT1 activity also resulted in VSMCs proliferation and neointimal hyperplasia (155). Interestingly, the *IRF1* promoter contains sequences that are recognized by both STAT1 and NF-κB. Detailed promoter analysis of differentially expressed inflammatory genes in coronary and carotid plaques in our recent data mining studies of atherosclerotic plaque transcriptomes predicted cooperative involvement of NF-κB, STATs, and IRFs (on ISRE, GAS, ISRE/GAS, ISRE/NF-κB, or GAS/NF-κB binding sites) in regulation of their expression in different cell types present in human atherosclerotic plaques (63, 156) (Figure 3). As such, the IRF-STAT-NFκB transcriptional mechanisms are a promising therapeutic target for the alleviation of atherosclerosis.

### IRF3 & 7

The IRF3/IRF7 heterodimer is widely implicated in viral infection, inflammatory diseases and plays an important role in promoting septic shock (157). Indeed, IRF3 and the closely related IRF7 are key regulators of IFN production induction and for this reason the majority of IRF3 and IRF7 KO mice studies were dedicated to understand their involvement in cell responses to pathogens, most of all viruses (Figure 3). Absence of IRF3 and IRF7 disrupts production of IFN-I and significantly increases pathogenesis (77). Mice deficient in both IRF3 and IRF7 exhibited an astonishing 1,000 to 150,000 fold higher level of viral RNA in their tissues after Dengue virus (DENV) infection than their WT counterparts (158). Shilte et al. showed that the lack of both IRF3 and IRF7 resulted in lethal infection in adult mice after exposure to West Nile virus (159). Moreover, a diminished IFN-I induced gene expression and higher viral burden was observed in response to Herpes simplex virus (HSV) or DENV infection in mice deficient in IRF3 and IRF7 (158, 160). HCV mutation studies have also shown that IRF3 and IRF7 are crucial for IFNλ2 and IFNλ3 transcription in HCV infected hepatocytes. Moreover, HCV is able to target and impair the expression of IRF3 via interaction with the basic amino acid region 1 of the HCV core protein. This action resulted in a lower expression of IRF3, and less dimer formation, enabling a persistent infection (161).

Following carotid artery injury, a significant decrease of IRF7 expression was observed in vascular smooth muscle cells (162). Mice overexpressing IRF7 in their smooth muscle cells specifically exhibited reduced neointima formation compared with their non-transgenic controls, while experiments with IRF7 deficient mice revealed an opposite effect (162). These results suggest that IRF7 is a modulator of neointima formation during atherosclerosis.

In addition, IRF7 regulated genes were highly expressed in breast cancer patients with a prolonged metastasis-free survival, suggesting diagnostic potential for this IRF family member (163). A more complete overview of involvement of IRF7 in cancer can be found in the review of Yanai et al. (164).

Systemic lupus erythematosus (SLE) is a common systemic autoimmune disease which affects a variety of organs, including skin, joints, lungs, kidneys, and nervous system (165). Many of the inflammatory cytokines released by leukocytes during SLE disease progression, such as IL-12, IL-6, IL-23, and IL-10 have ISRE sequences and are likely regulated by IRFs (166, 167). Indeed, IRF7 was found to be critical for the TLR9 pathway activation and the high production of IFN-I, observed in experimental SLE (168).

### IRF5 & 6

Recent studies in SLE and Rheumatoid Arthritis (RA), concluded that disease-associated atherosclerosis is mediated through IRF5. Likewise, mice deficient in IRF5 presented increased atherosclerosis and also exhibited hyperlipidemia, increased adiposity, and insulin resistance compared to WT controls (169). Moreover, IRF5 polymorphisms are implicated in several autoimmune diseases. In patients with SLE, genome-wide association studies showed that *IRF5* polymorphisms associated with disease risk (170–174). In RA, two polymorphisms of *IRF5* (rs2004640 GG and rs10954213 GG) revealed a protective effect against the risk of atherosclerosis and cardiovascular disease risk (175). Moreover, recently it was demonstrated that IRF5 is a target of the oncogene BCR-ABL kinase and restoration of IRF5 expression reduces Chronic myeloid leukemia (CML) cell proliferation (176).

IRF6 has recently been connected to TLR3 signaling in keratinocytes (49). TLR3 activation in these epithelial cells enhanced the expression of IFNβ, IL-23p19, IL-8, and CCL5. Silencing of IRF6 lead to an even higher expression of IFNβ, but a decrease in IL-23p19 (49). IRF6 has also been implicated in breast cancer, where it interacts with the mammary serine proteinase inhibitor (maspin), which is known to act as a tumor suppressor (177) (Figure 3). Moreover, IRF6 also has shown to bind the enhancer sequence of the p63 tumor suppressor gene (178).

### IRF4, 8 & 9

As IRF4, IRF8, and PU.1 are implicated in leukocyte development, it comes as no surprise that these co-activating complexes were found to be implicated in leukemia. IRF4 has been recognized to exhibit both oncogenic and tumor suppressor functions (179). Polymorphisms in the *IRF4* gene contribute to elevated IRF4 expression in cells from patients with multiple myeloma (180, 181). IRF4 deficient mice are characterized by normal distribution of B and T cells in earlier development with progressing lymphadenopathy throughout differentiation stages. IRF4 has been described as being essential for proper functioning and maintaining homeostasis of mature B and T cells (182) (Figure 3).

Proper activity of IRF8 is crucial for the regulation of apoptosis, mainly through activation of the Bcl-xL, Bax, and Fas genes in CML, although this anti-apoptotic potential of IRF8 is not limited to CML only (183, 184). Indeed, IRF8 deficient mice developed a syndrome resembling human chronic myelogenous leukemia (185). IRF8 has also been recognized as a key mediator of the cross-talk between cancer and immune cells (186). A Chinese study identified three SNPs in the *IRF8* gene (rs925994, rs11117415, and rs10514611) to be associated with susceptibility to tuberculosis (187). Together with the finding of Langlais et al., that in macrophages, IRF1/8 regulome transcripts appeared to be significantly enriched in genes commonly activated in tuberculosis infections (84), and the above mentioned involvement of IRF1 in tuberculosis, a significant role can be postulated e achfor IRF1/8 dimer activated gene expression in this disease too.

Analysis of IRF4,8 DKO mice fail to generate functional B cells due to arrest at the cycling pre-B-cell stage, and revealed that both transcription factors are relevant for DNA sequential rearrangement of immunoglobulins associated with B lymphocyte development (188). Inhibition of IRF4 accelerated c-Myc induced B-cell Leukemia in mice, suggesting its protective role by suppressing c-Myc gene transcription (189).

Studies in IRF9 KO mice models revealed that IRF9 and STAT1 are required for the production of IgG autoantibodies in the pristane-induced mouse model of SLE (190). The expression of NF-κB, along with TNFR1, and MCP-1 was increased locally in SLE associated skin lesions (191). Moreover, higher levels of NF-κB expression in SLE patients is linked to thrombosis formation [reviewed in (192)].

Based on their roles in these inflammatory diseases, IRFs and IRF-mediated transcriptional regulatory mechanisms represent interesting targets for therapeutic inhibition (Figure 3).

## Current IRF Inhibitory Strategies

There are several levels at which the activity of IRFs might be interrupted in a therapeutically advantageous manner (Figure 3). Indirect modulation might be achieved by targeting known activators and regulators of IRF expression as well as critical pathways downstream of IRFs. Most of the strategies currently known to modify the expression level of IRF proteins are based on the indirect effect of small natural or synthetic compounds. They act on TLRs or IFN receptors, by blocking ligand binding or preventing phosphorylation and downstream signaling (Figure 3, left side). Compounds may also inhibit formation of dimers or interaction of IRFs with other transcription factors or with co-activators. Blocking of IRF binding to target DNA sequences or preventing activation of transcription would be possible by direct binding of the inhibitory compounds to the IRF DBD or IAD domains (Figure 3, right side).

The inhibitory effect of several compounds on IRF1, 3 and IRF4 has been presented in relation to chronic inflammation and autoimmune disorders. The mechanisms of action of these compounds are mainly indirect, with the majority of them acting on components upstream of IRF signaling pathways (Figure 3). For example, Donepezil (DP) is an acetylcholinesterase inhibitor, approved by the FDA as a drug for alleviation of dementia in Alzheimer‘s patients. It exhibits inhibitory activity against IRF1 and its target matrix metalloproteinase13 (MMP13) involved in degradation of collagen, a root cause of osteoarthritis (OA). Thus, DP presents itself as a potentially effective therapeutic in OA treatment (193). VB-201, an oxidized phospholipid small molecule has been proposed as an effective atherosclerosis treatment agent *in vitro* and *in vivo*, due to its ability to directly bind to TLR2 and simultaneously inhibit IRF1 mediated signaling (194). A group of inhibitors specific toward either NOS2 (pyrrolidine dithiocarbamate, PDTC) or protein kinases (genistein—tyrosine kinase inhibitor; PD98059 and SB203580—MAP kinase inhibitors) has been described to modulate IRF1 expression (195). Leflunomide, a drug responsible for immunomodulation, also exhibits an inhibitory effect on MEK/MAP and thus on IRF1 (196). Several antipsychotic drugs, such as sertraline, trifluoperazine and fluphenazine were identified as specific inhibitors of the TLR3-IRF3 signal transduction pathway (197). Ruiz et al. characterized the anti-inflammatory role of flavonoids (apigenin, luteolin, genistein, 3′-hydroxy-flavone, and flavone) in relation to chronic intestinal inflammation. It revealed an inhibitory effect of these polyphenolic compounds on TNFα-induced NF-κB transcriptional activity and a subsequent decrease in *CXCL10* expression. Moreover, it was observed that luteolin and 3′-hydroxy-flavone induce IRF1 degradation (198). Anti-inflammatory and neuroprotective properties of luteolin have been confirmed in microglia. Luteolin exerted an inhibitory effect on NF-κB, STAT1 and IRF1, thus attenuating inflammatory responses of brain microglial cells (199). TNFα-dependent activation of IRF1 and transcription of the pro-inflammatory gene *CXCL10* is also repressed by the natural plant derivative Compound A (CpdA), which has been tested as a potent therapeutic agent for asthma (200).

Fungi and plants can also produce IRF3 modulating compounds. Zhankuic acid A (ZAA), a major pharmacologically active compound in fruiting bodies of *Taiwanofungus camphoratus*, acts as a JAK2 inhibitor that inhibits downstream signaling mediated by STATs and IRFs. Anti-inflammatory and hepatoprotective functions of ZAA have been evaluated in mice with acute hepatitis, leaving ZAA as a potential therapeutic agent for the treatment of inflammatory diseases (201). Thymoquinone (TQ) is a compound derived from black cumin, which indirectly inhibits IRF3 by affecting NF-κB and Activator protein 1 (AP1). Moreover, TQ targets the auto-phosphorylation of TBK1, an upstream key enzyme responsible for IRF3 activation (202). iNOS, an important inflammatory mediator, is linked to several inflammatory diseases and cancers. iNOS inhibitors (pinosylvin and BAY11-7082 – IKK inhibitor) have been shown to simultaneously block expression of IRF3 (203, 204). NSC95397 (2,3-bis-[(2-hydroxyethyl)thio]-1,4-naphthoquinone), as a multi-kinase inhibitor exhibits anti-cancer properties. This compound blocks activation of TNFα, AP1, and IRF3 in LPS-treated RAW264.7 cells and TRIF- and MyD88-overexpressing HEK293 cells (205).

More specialized IRF inhibitory mechanisms by direct disruption of transcription, nuclear translocation or DNA-binding have also been documented (Figure 3, right side). For example, the highly virulent bacterium *Francisella tularensis* uses its components to block NF-κB p65 activity, IRF1 translocation and binding of IRF1 and IRF8 to the Ets2 element in the promoter region of the IL-12 gene (206). IRF-dependent expression of IL-12 is also suppressed by adenylate cyclase toxin (CyaA) from *Bordetella pertussis* in DCs (207). Minocycline, a tetracycline antibiotic derived from fungi, experimentally used for treatment of many CNS disorders due to its anti-inflammatory properties have been shown to inhibit nuclear translocation of IRF1 (208). Also, Human Papilloma Virus core proteins have been recognized to mediate suppression of IRFs, in this case IRF1 synthesis at the transcriptional level. Subsequent repression of several ISGs, including Il-12 and Il-15, allows the virus to deceive the host organism and carry out an effective invasion (209).

Viruses have developed numerous strategies of direct interaction with IRFs to avoid and inhibit induction of innate immunity responses (Figure 3, left side). The human tumor-inducing herpesvirus, Karposi‘s sarcoma-associated herpesvirus (KSHV), successfully modulates the host IFN-mediated immune response. A unique evasion mechanism of KSHV reveals that this virus incorporates viral homologs of IRFs (vIRFs) to inhibit IRF7 DNA binding by blocking either the DBD or IAD of IRF7 (210) (Figure 3, right side). In a parallel study by Zhu et al., another inhibitory mechanism of KSHV has been reported. Namely, it demonstrated that ORF45 in association with virions interacts with IRF7 and subsequently blocks its phosphorylation and nuclear translocation (211). Further studies by this group revealed that ORF45 interacts with the inhibitory domain (ID) of IRF7 and keeps the protein in a closed, inactive form (212). Cai et al. reported that KSHV encoded Latency-associated nuclear antigen (LANA) evades MHC II presentation and blocks transcription of MHC II trans-activator (CIITA) by direct interaction with IRF4. The mechanism of inhibition is not fully understood, nevertheless it is documented that LANA blocks IRF4 DNA binding ability at promoter regions of CIITA (213). The group of Xing et al. reported that the HSV-1 encoded protein VP16 blocks the production of IFNβ by inhibiting NF-κB activation and preventing IRF3 from recruiting its co-activator CREB binding protein (CBP) (214). The Varicella-zoster virus (VZV) is known to antagonize the IFNβ pathway in the IRF3 branch. It has been demonstrated that VZV immediate-early protein ORF61 mediates degradation of IRF3 via direct interaction. Interestingly, it has been shown that ORF61 only targets the phosphorylated form of IRF3 and not the unphosphorylated IRF3 in uninfected cells (215). Another example is the HCV, which targets IFN signaling pathways through a mechanism based on the inhibition of IRF3 phosphorylation and activity by non-structural viral proteins (216). It was shown that NS3/4A, a serine protease, can successfully block IFNβ production by suppressing RIG-I and IRF3 activation (217). Moreover, the HCV NS5A protein was able to block IRF7-mediated IFNα promoter activation, which might be in part responsible for the successful establishment of chronic HCV infection (218).

Finally, molecular biology tools of gene silencing, including RNAi technology and ncRNA, have been employed in IRF targeting for cancer treatment (Figure 3, right side). High expression of *IRF1* and *IRF2* have been observed in human leukemic TF-1 cells. The group of Choo et al. developed a novel screening protocol in order to identify effective siRNAs targeting IRF2 in leukemic cells (219). IRF4 activity has been linked to a number of germinal center (GC) and post-GC B lineage subset malignancies (179). In 2010 microRNAs essential for plasma differentiation mediated by IRF4 were identified. Moreover, microRNA 125b was characterized to inhibit B cell differentiation in GCs (220). Another group reported that expression of *IRF4* inversely correlated with microRNA (miR)-125b in multiple melanoma patients. Positive inhibitory effects of this synthetic microRNA have been confirmed *in vitro* and *in vivo*, leaving IRF4 as an interesting multiple myeloma therapeutic target (221).

## A Direct IRF-Targeting Strategy to Identify Specific- and Pan-IRF Inhibitory Compounds

Despite the large number of described compounds indirectly modulating IRF activity, there are still no effective strategies based on direct inhibition. None of the strategies studied so far have relied on the use of directly interacting compounds, which would affect the IRF protein structure. Moreover, no potential inhibitory binding sites in IRF-DBD or IAD have been proposed in the existing literature. The direct modulation of IRFs has not been attempted previously due to several reasons. Above all, to overcome possible variations between conformational differences under physiological vs. *in silico* conditions, we considered both apo- and holo- forms of IRF DBD in this approach. There are two types of IRF-DBD structures deposited in PDBe or RCSB PDB; inactive cytoplasmic free forms and active nuclear DNA bound forms. Under physiological conditions IRFs undergo major conformational changes when they transform from inactive to the active state. To efficiently inhibit IRFs, it is essential to identify compounds which would bind to the inactive form and block conformational changes and DNA binding. We propose the IRF DNA binding site as the most promising active site for inhibition. Moreover, we believe that the good quality models presented here supported by our previously described pipeline approach CAVS, which combines comparative *in silico* docking to the IRF-DBD with *in vitro* validation of potential inhibition will prove to be successful in the search for effective inhibitory compounds. Only after thorough *in vitro* validation we will be able to prove effectiveness of *in silico* selected compounds as potential IRF inhibitors, as well as asses their possible cytotoxicity. Taken together, an in-depth understanding of the IRF protein structure and the mechanisms involved in the binding of these transcription factors to DNA will allow the development of potent and effective inhibition strategies.

**Figure 4:**
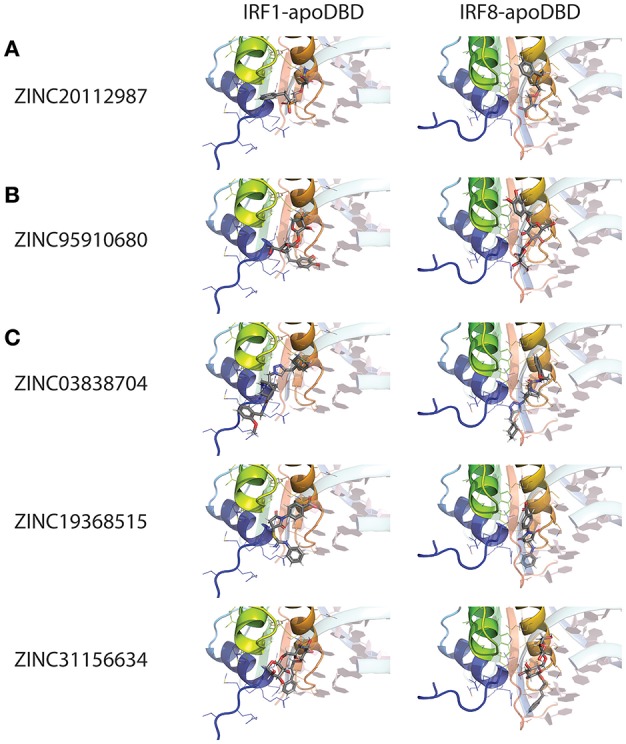
Binding conformations of pre-selected compounds from Natural Products ZINC database subset in different IRF DNA-binding domains. **(A)** Top-scored IRF1-specific binding compounds in apo-DBD of IRF1 and IRF8; **(B)** Top-scored IRF8-specific binding compounds in apo-DBD of IRF1 and IRF8; **(C)** Top-scored pan-IRF1/8 binding compounds in apo-DBD of IRF1 and IRF8. Graphical representation, that has been used, describes in detail binding mode of top-scored conformation of the inhibitor in the active pocket of apoIRF-DBD. dsDNA fragment of the respective holoIRF-DBD/IRE complexes superimposed on the apo-form implicates the position of selected target cavity for inhibitory compound. The best binding conformation of each potential inhibitor is shown in stick representation (carbon—gray; oxygen—red; nitrogen—blue; phosphorus—orange and hydrogen—white). IRF1 and IRF8 apo-DBDs are in the cartoon representation with visible secondary structure, multi-colored with amino acid side chains that interact with compounds shown as lines. dsDNA fragment of the respective IRF-holo-DBD/IRE complexes is shown in 60%-transparent cartoon representation and colored in pale-cyan with nucleobases colored in light-pink. Ligand docking results were obtained using Surflex-Dock 2.6 software.

For this reason, we generated 3D structure models for IRF1, 2 and 8 DBDs (10), presented in two distinct conformations essential to the function of IRFs. Namely, non-DNA-bound cytoplasmic conformations known as apo-forms, and DNA-bound nuclear conformations or holo-forms. In our effort to identify specific inhibitors for different STATs, we developed a five-step comparative virtual screening tool, CAVS (222). Subsequently, we utilized this *in silico* screening method to identified potential specific IRF1-DBD and IRF8-DBD inhibitors (10). The basic assumption of the system is the adaptation of two main selection criteria to evaluate virtual screening results: Comparative Binding Affinity Value (CBAV)—a measurement of the binding quality between different IRFs, and Ligand Binding Pose Variation (LBPV), which reflects compound binding specificity (222). The LBPV ratio (from 0 to 1) represents the conformational conservation of all 20 output conformations obtained from docking. Previously we presented top-scored IRF1-specific and IRF8-specific inhibitors in apoDBD of IRF1, IRF2, and IRF8, where IRF2, as a closest correlate to IRF1, was used as a control for comparison. CBAV-IRF(1-2), CBAV-IRF(1-8), and CBAV-IRF(8-2) were determined to compare the binding affinities between IRF1, IRF2, and IRF8 for both compounds. Consequently, we obtained 60 top hits for IRF1-DBD and 7 top hits for IRF8-DBD (data not shown). The compounds were ordered based on descending CBAV-IRF(1-2) and CBAV-IRF(8-2) values, which allowed to select the most potent IRF1 and IRF8 targeting molecules displaying at the same time low affinity to the IRF2-DBD control. Here we present the top 3 IRF1-specific and IRF8-specific compounds (Table 1) and the graphical representation of ZINC20112987 and ZINC95910680 fitted into the binding cavities of IRF1 and IRF8 in a new graphic design mode (Figures 4A,B). High CBAV values (>3) of compounds ZINC20112987 (4, 82), ZINC08623925 (4, 42), and ZINC20112989 (4, 25) confirm their high binding affinity toward IRF1 and not IRF8. Analogously, CBAV values of compounds ZINC20112987 (4, 26), ZINC08623925 (3, 36) and ZINC20112989 (3, 01) point to their possible specificity toward IRF8 (Table 1). For example, ZINC20112987 has IRF1-LBPV of 0.75 meaning high conformational conservation toward IRF1-DBD and subsequent significantly lower IRF8-LBPV. Likewise, ZINC95910680 (IRF8-LBPV = 0.85) displays high conformational conservation toward IRF8-DBD, but low conservation within IRF1-DBD (Table 1).

**Table 1:** Potential pan-IRF1/8-DBD; IRF1-DBD specific and IRF8-DBD specific inhibitors.

**Ligand**	**IRF1-BS**	**IRF8-BS**	**CBAV**	**IRF1**	**IRF8**
				**LBPV**	**LBPV**
**pan-IRF1/8-DBD^**a**^**					
ZINC03838704	7.736	7.7377	−0.0017	0.4/0.3	0.4/0.35
ZINC19368515	7.4603	7.4238	0.0365	0.2/0.25	0.3/0.35
ZINC31156634	8.514	8.4656	0.0484	0.25/0.15	0.5/0.15
**IRF1-DBD SPECIFIC^**b**^**					
ZINC20112987	12.6694	7.8452	4.8242	0.75	0.4
ZINC08623925	11.0354	6.611	4.4244	0.9	0.4
ZINC20112989	12.6045	8.3569	4.2476	1.0	0.8
**IRF8-DBD SPECIFIC^**c**^**					
ZINC95910680	7.4184	11.6812	4.2628	0.3	0.85
ZINC35465373	4.7842	8.144	3.3598	0.2	0.75
ZINC85542529	3.6799	6.6869	3.007	0.35	0.7

*Comparative docking characteristics: pgeom algorithm, CBAV and LBPV of top selected compounds from Natural Products screen bound to IRF1- and IRF8-DBD domain. In silico calculations were performed by Surflex-Dock 2.6. BS, binding score; CBAV, comparative binding score value; LBPV, ligand binding pose variation*.

a *CBAV(pan-IRF1/8) = BS(IRF1) – BS(IRF8)*.

b *CBAV(IRF1spec) = BS(IRF1) – BS(IRF8)*.

c *CBAV(IRF8spec) = BS(IRF8) – BS(IRF1)*.

Moreover, by adapting the comparative docking and selection of STAT inhibitory compounds, CAVS (223), we recently re-evaluated previously considered STAT3-specific inhibitors STATTIC and STX-0119 as pan-STAT1/2/3 inhibitors in vascular inflammation (224). Analysis of the corresponding total binding score values (BS) and CBAVs of STATTIC and STX-0119 calculated for each individual STAT, points to their equal binding affinities for STAT1, STAT2, and STAT3 (222). In the same study, we described a novel pan-STAT1/2/3 inhibitor, C01L_F03, with similar characteristics (225). We proposed that this novel class of inhibitors could be implemented in a multi-STAT inhibitory strategy with great promise for the treatment of Cardiovascular diseases (CVDs) (225). Accordingly, blocking of IRF-DNA binding by IRF-specific or pan-IRF inhibitors presents itself as a promising therapeutic tool to combat a wide range of immunological diseases. In case of disorders where only one specific member of the IRF family is involved in disease development, usage of compounds specific toward this particular IRF would be the most suitable. Such an IRF-specific inhibitor based approach, could be applied for many previously described infectious diseases and cancers. Nevertheless, many disorders are dependent on aberrant interaction of two or more IRFs at the same time. A representative example of an autoimmune disease where the use of a pan-IRF inhibitor could be advantageous is SLE, in which combined action of IRF5 and IRF7 has been documented (168, 169). A similar strategy could be applied for numerous chronic inflammatory diseases, such as RA or atherosclerosis. Several studies (169, 226, 227) pointed to the role of IRF1 as well as IRF5 in OA or RA, while IRF1, 5, 7, and 8 are recognized as key factors contributing to development and progression of atherosclerotic plaques (156, 169, 175).

STAT family members together with IRF1 and IRF8 were identified as key mediators of inflammation associated with CVDs. Therefore, going a step further, we used described 3D models of IRF1 and IRF8-DBD apo-forms, as the molecular targets for a virtual screening strategy, in order to identify pan-IRF1/8 inhibitors. Herein, we present a list of the top 3 compounds with a high inhibitory potential toward both IRF1 and IRF8 (Table 1 and Figure 4C). The five-step docking procedure, subsequently resulted in a list of 20 optimized conformations for each selected compound, with supporting BS, CBAVs and LBPVs for each IRF. Table 1 shows the top IRF1-BS and IRF8-BS of ZINC03838704, ZINC19368515 and ZINC31156634, as well as CBAV-IRF(1-8). In an ideal situation, the value of CBAV parameter for pan-inhibitors is equal or close to 0. After analysis of corresponding CBAV (−0.01 – 0.04) values it becomes clear that presented compounds exhibited nearly identical binding affinity to the IRF1 and IRF8 DBD. Compounds are presented according to ascending CBAV values, which allowed to select the most potent pan-IRF1/8-DBD targeting molecules. Figure 4C illustrates the top scored conformation of ZINC03838704, ZINC19368515 and ZINC31156634 compounds in IRF1- and IRF8-DBD, as representative pan-IRF1/8-DBD inhibitors. While for IRF-specific inhibitors one dominant pose represented the compounds' conformational tendency, for pan-IRF inhibitors it was common that two dominant binding conformations oriented in opposite directions were observed, which results in two LBPV values calculated. LBPV in the range of 0.8; 1.0 represented low conformer diversity and significant binding specificity of the compound to IRF-DBD, whereas the range of 0.0; 0.2 denotes high conformer diversity and poor binding specificity. Low-throughput *in vitro* cell-based multiple activation and IRF inhibition should be used to validate the effect of pre-selected inhibitory compounds on cytokine-induced IRF action and target gene expression in different cell types.

## Diagnostics, Therapeutics & Future Perspectives

### IRFs in Diagnostics

**Figure 5:**
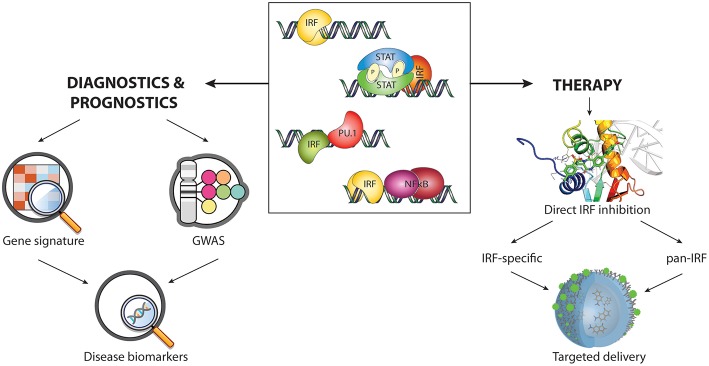
Therapeutic, diagnostic, and prognostic space occupied by IRFs and IRF-mediated transcription regulatory mechanisms. IRF-containing complexes involved in transcriptional regulation, including IRF homodimers, STAT/IRF, PU.1/IRF, and NF-κB/IRF (central), can serve as diagnostic and therapeutic targets in different ways. The analysis of IRFs transcriptional mechanisms together with GWAS and characterization of gene expression signatures might provide disease diagnostic and prognostic markers (left side). Novel IRF-specific and pan-IRF inhibitors combined with an appropriate delivery system have the potential to be effective therapeutic agents for numerous immune related diseases (right side). We propose nanoparticles as promising tool for IRF inhibitory compound administration.

IRFs have an important role in various diseases. In recent years their clinical relevance was established by genome-wide association studies (GWAS). Applying genome-wide SNP association studies, it was demonstrated that IRF4 is strongly associated with susceptibility to Chronic lymphocytic leukemia (CLL), with risk loci identified at 6p25.3 (rs872071, IRF4) (228). IRF5 and IRF7 alleles rs2004640 and rs1131665 predispose to the development of SLE (173, 174, 224). Acknowledging the implications of IRF4, IRF5, and IRF7 polymorphisms and aberrant expression in autoimmune diseases like SLE, RA and cancer, prognostic screening could provide insights in disease severity (Figure 5).

Changes in IRF expression could be a prognostic factor in several human diseases. For instance, IRF1, IRF4, and IRF8 are significantly downregulated in failing human hearts compared with healthy controls (229, 230), whereas IRF3 is profoundly upregulated in the hearts of patients with dilated or hypertrophic cardiomyopathy (231). IRF1 and IRF2 expression is associated with prognosis and tumor invasion in hepatocellular carcinoma (HCC). Supporting this notion, the IRF2/IRF1 ratio positively correlated with tumor metastatic potential in the metastatic model of HCC cell lines—HCCLM3 (232). Hence, IRF expression can be used as viable prognostic markers in SLE and several types of cancer (Figure 5).

Not only IRF expression itself, but downstream ISG expression provides interesting markers for diagnostic use. With the demonstrated variety in IRF-mediated transcriptional regulatory mechanisms implicated in diseases, leading to the upregulation of specific subsets of ISGs, several applications can be envisioned (Figure 5). Indeed, specific subsets of ISGs are already being proposed for use in assays for the prediction of recurrence risk in patients with colon cancer and assays assessing the risk of transplant rejection (233, 234). In previous work our group identified a 72 gene “plaque signature” that predominantly consisted of STAT1 and IRF8-target genes which could be of use as a novel diagnostic tool to monitor and diagnose plaque phenotype in human atherosclerosis (156).

### IRF in Therapeutics

Dysregulation of IRF function is critical in the development of immune system-originated diseases. Therefore, investigating the regulatory mechanisms mediated by IRFs and modulating IRFs expression might be crucial for disease treatment (Figure 5).

So far, IRFs have not been pursued as drug targets in terms of direct and selective inhibition. Current IRF inhibitory strategies are mainly limited to indirect modulation of their expression and function. Only direct inhibition strategies, which target IRFs transcription by siRNA or miRNA have been employed. Preventing IRF binding to DNA could serve as another potential therapeutically advantageous way to inhibit IRFs. An in-depth understanding of the IRF protein structure and the mechanisms involved in the binding of these transcription factors to DNA will allow the development of effective inhibition strategies. Moreover, the fact that many IRFs require a binding partner, such as PU.1, to effectively contact DNA can be used to develop a potent inhibitory system for IRFs. In addition, formation of homo- and heterodimers or cooperative DNA binding with co-activators, both promoted by the IAD in the C-terminal region can be directly blocked by inhibitory compounds.

We postulate that successful targeting of IRF-DBDs using small-molecule inhibitors provides hope that IRFs can be “attacked” directly and used for the treatment of IRF-dependent disorders and malignancies. Considering the similarities and differences between the individual IRFs, in particular two directly modulating IRF DNA binding strategies can be proposed (Figure 5). The first approach would be based on the specific inhibition of IRF responsible for the development of the disease. Selective targeting of the IRF-DBD could lead to overcoming viral or bacterial infections as well as cancers. The second strategy would be designed to trigger a pan-IRF effect and inhibit two or more causative IRFs e.g., in SLE treatment. In addition, existing protein-DNA and protein-protein interfaces of human IRFs can be screened for potential cavities selectively binding inhibitory compounds.

### Future Perspectives

In this review, we have summarized the current knowledge of the different IRF-mediated transcriptional regulatory mechanisms and how they reflect the diverse functions of IRFs in homeostasis and in TLR and IFN signaling. IRFs orchestrate expression of distinct subsets of ISGs via dimer formation, their involvement in transcriptional complexes, and co-binding with other transcription factors. ISG subset expression, as well as the expression of IRFs or the SNPs they contain might be exploited in future diagnostic arrays for the assessment of disease progression of a wide variety of (auto)immune diseases and cancer.

Several STAT inhibitors, including synthetic small compounds, natural products and oligonucleotide decoys, in recent (pre)clinical trials prove that strategies targeting transcription factors might find their way to the clinic in the near future. Therefore, we postulate that successful targeting of IRF-DBDs using small-molecule inhibitors provides hope that IRFs can be “attacked” directly and used for the treatment of IRF-dependent disorders and malignancies (Figure 5).

*In vitro* and *in vivo* validation of IRF inhibitory compounds has to prove their hypothesized effectiveness, as well as assess potential cytotoxicity before these products can move to clinical studies. Another challenge for the use of inhibitory strategies in therapeutics is the administration of such compounds. Systemic administration of IRF inhibitors is undesirable, because of possible unforeseen side effects. Therefore, either local injection/release or targeted administration with labeled compounds will be more effective as inhibition therapy. Nanotechnology might offer novel ways of drug administration (Figure 5). Antibody-conjugated nanoparticles have previously been used experimentally (235), and already several studies with nanoparticle based administration of inhibitory drugs have been published, such as inhibition of PI3K or the apoptotic regulator protein survivin in several types of cancer (236, 237). The results reported in such studies strengthen the feasibility of a nanomedicine targeted approach to IRF inhibition.

## Author Contributions

AA and BK were equally involved in concept development, writing and editing the manuscript. MS generated 3D models of IRFs DBD domains and performed *in silico* analysis and described the results. AM took part in figures preparation and concept development. AP-B, MP-G, and JW participated in development of the concept and critically evaluated and edited the manuscript. HB developed the concept and was involved in writing and editing the manuscript and coordinated input from all co-authors.

### Conflict of Interest Statement

The authors declare that the research was conducted in the absence of any commercial or financial relationships that could be construed as a potential conflict of interest.
